# Basic science of electronic cigarettes: assessment in cell culture and *in vivo* models

**DOI:** 10.1186/s12931-016-0447-z

**Published:** 2016-10-07

**Authors:** Pieter S. Hiemstra, Robert Bals

**Affiliations:** 1 Department of Pulmonology, Leiden University Medical Center, Leiden, The Netherlands; 2 Department of Internal Medicine V – Pulmonology, Allergology and Critical Care Medicine, Saarland University, D-66421 Homburg, Germany

## Abstract

Electronic cigarettes (e-cigarettes, ECIGs) were introduced into the market a decade ago as an alternative to tobacco smoking. Whether ECIGs are safe and whether they qualify as smoking cessation tool is currently unknown. Their use has markedly expanded in that period, despite the fact that potential toxic effects of the vapour created by the e-cigarette and the nicotine-containing cartridge fluid have been incompletely studied. Marketing targets diverse groups including older smokers but also young people. Whereas the adverse health effects of nicotine inhaled by users of ECIGs has been well documented, less is known about the other components. An increasing number of *in vitro* and *in vivo* studies demonstrate a range of adverse effects of both the vapour created by ECIGs as well as the nicotine-containing fluid. Importantly, these studies demonstrate that toxicity from ECIGs, although this may be less than that caused by tobacco products, not only arises from its nicotine content. Furthermore, there are no data on the long-term consequences of ECIG use. The wide range of ECIG products available to consumers and the lack of standardisation of toxicological approaches towards ECIG evaluation complicates the assessment of adverse health effects of their use. Here we review the current data on preclinical studies on ECIGs describing their effects in cell culture and animal models.

## Background

The use of electronic cigarettes is steadily increasing and has drawn the attention from law makers, the tobacco industry, health organizations, researchers, smokers and non-smokers [1]. Whereas electronic cigarettes (ECIGs) are promoted as a safer alternative to tobacco smoking and may potentially help reduce tobacco consumption, they might also need to be considered as new and potentially harmful products causing adverse health effects. Furthermore, there is concern that use of ECIG by e.g. young non-smokers may induce nicotine-dependency. Therefore, pros and cons of ECIGs are a central topic in a vigorous debate, which is furthermore complicated by the fact that the current body of data is limited and does not allow to definitely answer the question whether ECIGs are good or bad [2]. PubMed currently (5/2016) lists 2896 hits on the search topic “electronic cigarette” with a high proportion of articles with no primary data but reviewing the subject or giving an opinion.

**Fig. 1:**
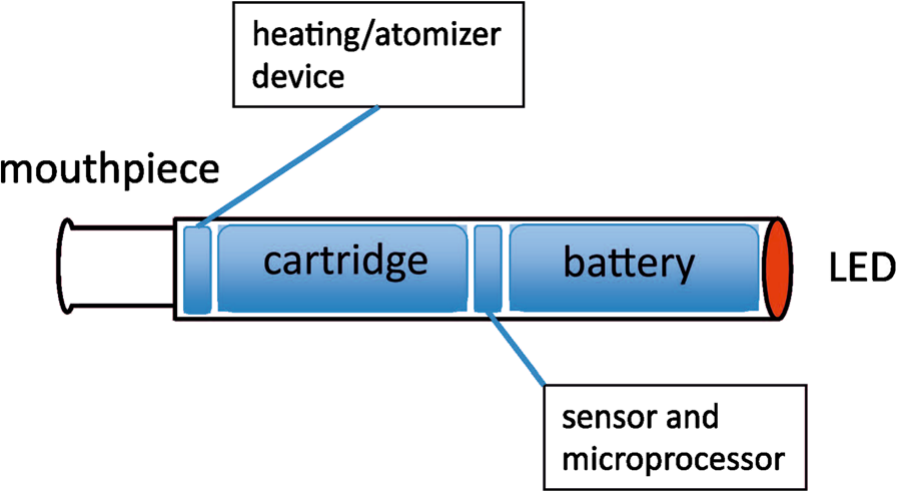
Electronic cigarette. The cartridge contains a fluid with nicotine, flavours, propylene glycol and water. The heating/atomizer heats the content of the cartridge to create a vapour that can be inhaled through the mouthpiece. The (pressure) sensor detects the airflow when the smoker inhales, and signals the microprocessor to control the heating element and the LED tip. This tip lights up when the smoker inhales to mimic the glow of a burning cigarette. A (rechargeable) battery provides the power

The first generation of ECIGs or electronic nicotine delivery systems (ENDS) were introduced on the market in the European Union in 2006 and in the United States of America in 2007. ECIG differ from conventional tobacco cigarettes because they vaporize a heated fluid instead of burning tobacco. This ECIG liquid is composed of a variable combination of nicotine, propylene glycol, glycerol, water, and various flavours. This mixture is heated by an electronic device to generate a vapour that is inhaled (Fig. 1). Based on this definition, tobacco heating systems developed by the tobacco industry as an alternative to conventional tobacco combustion are not considered as an electronic cigarette and are therefore not discussed in this review. There has since been substantial development in the design and performance of ECIGs, including mixing and matching options for creating individual ECIG liquids, temperature regulation, increased delivery of nicotine, and currently fourth generation ECIG are available.

ECIGs have been proposed as a safer alternative to conventional cigarettes, but as outlined above there is concern about the toxic properties of EC. Importantly, at present there is no regulation regarding the characteristics of EC emissions or their effects on biological systems. This is important, especially in view of their safety upon long-term use. In this review we focus on the results from studies aimed at investigating potential toxic effects of ECIGs using preclinical models such as cell culture and animal models. Whereas such preclinical studies are often criticized because they may not fully predict the response of the human body to the exposure, animal testing is still the cornerstone of regulations around toxicology testing and in vitro models are only slowly being accepted as an acceptable alternative.

A large number of ECIG liquids is commercially available. In many cases, the quality of the production process of the components is inadequately documented. Glycerol and propylene glycol are small chemicals that are liquids at room temperature and that are widely used as food additive and in pharmaceutical applications [3]. Toxicology studies revealed low toxicity, while no systematic data are available on chronic inhalation. The effect of nicotine has been widely studied and it is evident that this substance has a variety of harmful properties, including being highly addictive and supporting cancer growth [4, 5]. In addition to these substances, a huge number of flavours are included in ECIG that are not subject to any regulation. Several studies highlight the complexity and potential harmfulness of these additives [6–8]. Whereas these flavours are widely used as food additives, their effects upon inhalation are largely unknown. The importance of this gap in our knowledge is illustrated by the observation that diacetyl and diacetyl-containing flavours that are used in butter-flavoured microwave popcorn cause bronchiolitis obliterans upon inhalation [9]. Importantly, diacetyl is present in many sweet-flavoured ECIG-liquids at relevant concentrations [10]. Furthermore, heating of ECIGs at too high temperature using a high voltage leads to generation of highly toxic formaldehyde [11].

There are no long term data on the effects of ECIG liquids or vapour on the human lung. As a consequence, it is unknown whether chronic ECIG consumption might cause disease and if yes, what type of disease. Preclinical studies in cell culture and animal models help to estimate potential toxic effects, notwithstanding the notion that these data have to be interpreted carefully. One specific issue is the lack of standardization. For preclinical studies on health effects of tobacco, standardized protocols have been developed for exposure machines [12]. Currently, there is a lack of standardization for preclinical analysis of ECIGs. The development of such a standardization is necessary as the complexity of the exposure systems (with variation of ingredients, vaping profiles, heating temperatures, use of tubing and exposure setup) does currently not allow comparison and generalisation of the outcomes of the various studies.

### Analysis of effects of ECIGs on cell cultures

An increasing number of studies is reporting on effects of ECIGs on cultured cells, studies that were initiated to gain insight into the biological and toxicological effects of ECIGs. Different approaches were used to investigate these effects, and both effects of ECIG-liquids, as well as effects of vapour generated by ECIGs and inhaled by users, were investigated. Effects of ECIGs were evaluated using a wide range of target cells. Some studies focussed on the cells that are in direct contact with the inhaled ECIG-vapour, such as airway epithelial cells [8, 13–20]. This is highly relevant, since the airway epithelium requires specific attention since it is the first and largest body surface exposed to smoke derived from an ECIG or tobacco cigarette. In these studies, discussed in detail in the next paragraphs, both ECIG vapour, ECIG liquid and ECIG vapour extracts were used. The potential systemic and other consequences of ECIGs were investigated by studying the effect of ECIG-vapour or liquid on a broader range of cell types including human fibroblasts [8, 21, 22], murine fibroblasts [23], endothelial cells [24], vascular smooth muscle cells [25], rat Kupffer cells [26], human embryonic stem cells [21], neutrophils [27], and murine neural stem cells [21].

There are also major differences between studies in the use of tumour cell lines, immortalized cell lines and primary cell lines. This is especially important when studying exposures of airway epithelial cells that are well differentiated and composed of various cell types including basal cells, mucus-producing goblet cells, ciliated cells and club cells [28]. Primary airway epithelial cells show this differentiation when cultured at the air-liquid interface, whereas most immortalized or tumour cell lines do not. Therefore, it can be argued that for studying the effect of aerosols on epithelial cells, the use of primary airway epithelial cells and air-liquid interface (ALI) culture and exposure systems is best suited. Nevertheless, in inhalation toxicology the use on non-differentiated tumour of immortalized cell lines is widespread because these cells are easier to handle, do not show inter-donor differences (because they are derived from one donor), and have an extended life span, thus increasing their availability.

Thus far, only one study has used primary human airway epithelial cells that were differentiated at the ALI and exposed to ECIG-vapour at the ALI [29]. This study from British American Tobacco, a company that produces both tobacco cigarettes and ECIGs, showed that ECIG-vapour exposure did not result in cytotoxicity or decrease in epithelial barrier activity as assessed by transepithelial electrical resistance (TEER), in contrast to exposure to whole cigarette smoke. Two other studies investigated the effect of ECIG-vapour on airway epithelial cells using non-differentiated primary airway epithelial cells, showing reduced viability [14, 15] and increased oxidative stress [15], whereas Lerner et al. showed that exposure of the airway epithelial tumour cell line NCI-H292 causes increased production of IL-6 and IL-8 [8]. In one of these studies, the effect of ECIG-vapour on non-differentiated primary bronchial epithelial cells, a new immortalized bronchial epithelial cell line with differentiation potential (CL-1548), and the A549 cell line was compared [14]. The results showed that A549 were least susceptible to the aerosol when using cell viability as a read-out, whereas primary bronchial epithelial cells were most susceptible and CL-1548 showed intermediate sensitivity. Interestingly, despite the fact that primary bronchial epithelial cells and CL-1548 showed apparent comparable differentiation capacity, for the exposure experiments non-differentiated cultures were used. This study also confirmed that the toxicity resulting from ECIG vapour exposure was markedly lower than that resulting from tobacco smoke. This was also the conclusion from another study using exposure of the A549 alveolar epithelial tumour cell line to ECIG-vapour using cell viability and pro-inflammatory cytokine release as a read-out [19].

Three studies reported on the use of an aqueous ECIG-vapour extract. One study used the immortalized bronchial epithelial cell line BEAS-2B and showed that ECIG-vapour extract causes protein aggregation due to inhibition of autophagy, resulting in oxidative stress, apoptosis and senescence [17]. This mechanism has been proposed to contribute to COPD development and progression, and thus may also contribute to adverse health effects of EC. Another study used the A549 cell line, and showed that ECIG-extract decreased cell viability but to a far lesser extent than cigarette smoke extract [20]. Finally, an ECIG-vapour extract caused reduced cell viability and DNA strand breaks in the keratinocyte cell line HaCaT and in head and neck squamous cell carcinoma cell lines [18].

Other studies investigated the effect of ECIG-liquid on airway epithelial cells. Whereas application of ECIG-liquid to non-differentiated primary airway epithelial cells caused an increase in IL-6 production and rhinovirus infection, accompanied by a decrease in production of the innate host defense mediator SPLUNC1 [13], ECIG-liquid application to ALI-differentiated primary epithelial cells caused a shift in the metabolome [16]. The analysis of the effect of ECIG flavouring additives is complicated by the increasingly large number of companies that offer these liquids. A screening approach has been used to test multiple ECIG liquids on the epithelial cell line 16HBE14o- and subsequently in well-differentiated mouse epithelium [30]. A number of liquids with toxic potential were identified and the chocolate flavouring 2,5-dimethypyrazine was identified to activate CFTR in epithelial cells. Another study investigated the interaction between ECIG liquids and neutrophils and found that exposure of neutrophils to extracts of the ECIG vapour induced a pro-inflammatory response characterized by induction of CD11b, CD66b, MMP-9 and CXCL8 [27].

In summary, these studies that used a variety of approaches show adverse effects of ECIG vapour and liquid on primary airway epithelial cells and tumour cell lines, and other epithelial cell lines, that ranged from reducing viability, an increase in production of inflammatory mediators and oxidative stress, to reducing antimicrobial defences and pro-carcinogenic events. Only one study did not observe adverse effects, but only assessed cell viability and epithelial barrier function as read-out [29]. Interestingly, in four of the studies showing adverse effects, the specific contribution of nicotine to these effects was investigated, and it was demonstrated that these effects were not only mediated by nicotine and even some times largely independent of nicotine concentrations [13, 15, 18, 19]. This is in line with the results from a study on the effect of ECIG-liquid on human gingival fibroblasts [22].

These studies on epithelial cells and a variety of other cell types demonstrate that ECIG-vapour and ECIG-liquid may be less toxic than cigarette smoke, but do cause marked adverse effects on a variety of parameters in various relevant cell types including airway epithelial cells. The studies are somewhat difficult to compare because of differences in cell types, exposure systems and ECIG brands investigated. In addition, the lack of uniformity in generating EC aerosols also hampers interpretation of these studies [7]. Future studies are needed to harmonize approaches to investigate potential harmful effects on cell cultures.

### Application of ECs in animal studies

Animal studies have been extensively used to study the effect of exposure to cigarette smoke in development of lung diseases such as chronic obstructive lung disease (COPD) or lung cancer [31]. While these models have expanded the knowledge about disease mechanisms, there also was criticisms whether these results can be translated into clinical practice [32]. It is also a challenge to compare the results between different species or experimental exposure systems used in various setups. Nevertheless, animal models might be a valuable tool to learn about the potential long term outcomes of the exposure to ECIGs. A few studies exist that applied ECIG solutions or aerosols to animals in experimental models.

Neonatal mice were exposed to ECIG for the first 10 days of their life and were found to have modestly impaired lung growth, alveolar cell proliferation, and total body weight [33]. The whole body exposure system comprised a commercial ECIG, from which an aerosol was generated by a pump. In a murine model of asthma, which was induced by systemic sensitization to ovalbumin, the application of diluted ECIG solution increased airway inflammation including an increase in eosinophils levels of Th1-cytokines IL-4, IL-5 and IL-13, and OVA-specific IgE, and worsened hyperresponsiveness [34]. In a recent paper, mice were exposed to aerosolized phosphate-buffered saline, nicotine-free or nicotine-containing ECIG solutions [35]. Exposure to inhaled nicotine-containing e-cigarette fluids triggered effects normally associated with the development of a COPD-like tissue damage in a nicotine-dependent manner.

Cigarette smoke is known to inhibit the innate host defense of the lung [36]. One study investigated the effect of exposure to ECIG vapour for two weeks and showed an increased susceptibility to infection with influenza A and *Streptococcus pneumoniae* [37]. The effect of ECIGs was linked the oxidative stress and impaired phagocytosis. In this study, the animals were whole-body exposed to an aerosol of commercial ECIGs applied to a classical smoking machine while monitoring aerosol exposure and cotinine levels in the animals. The generation of oxidative stress by ECIG was studied by exposing C57B/6 mice to aerosols from commercial ECIG devices using a standard smoke exposing system [8]. ECIG exposure resulted in increased levels (IL-6, MCP-1, IL-1α, IL-13) and decreased glutathione levels. There was no comparison to conventional cigarettes.

The psychological and behavioural effects of ECIGs were studied using whole-body exposure to cigarette smoke or ECIG vapour, followed by a series of biochemical and behavioural studies. The results showed that nicotine-containing ECIG vapour induces addiction-related neurochemical, physiological and behavioural changes [38]. The offspring of the pregnant mice, which were exposed to nicotine-containing ECIG liquid, showed significant behavioural alterations. This indicated that exposure to ECIG components in a susceptible time period of brain development could induce persistent behavioural changes [39].

## Conclusions

There is currently a limited amount of data on the effect of ECIGs preclinical models. The main findings can be summarized as followed:

- There is a lack of standardization of exposure systems making it difficult to compare exposures, models and outcomes. The diversity of ECIG products, the complexity of ingredients, and vaporizing conditions contributes to the variability of preclinical ECIG studies.
- ECIG vapours has adverse effects on both cultured cells and living animals. Various outcomes have been measured in models. ECIGs induce inflammation, augment the development of allergic airway inflammation in asthma models, change the behaviour of animals, and suppress pulmonary host defense.
- Based on most parameters investigated in in vitro and in vivo studies, ECIG appear less harmful than tobacco cigarettes. The long term adverse health effects of ECIG use in humans cannot be predicted from the currently available data.

The analysis of health effects of ECIGs has only just begun despite the fact that ECIGs have been on the market for a decade. In view of the importance of insight into the long term consequences of ECIG use, it is important to realize that currently available information from in vitro and in vivo models may not provide final answers but certainly contribute to the knowledge on this novel product.

## Abbreviations

ECIGs: Electronic cigarettes, e-cigarettes
ENDS: Electronic nicotine delivery systems
